# Delivering Diabetes Education through Nurse-Led Telecoaching. Cost-Effectiveness Analysis

**DOI:** 10.1371/journal.pone.0163997

**Published:** 2016-10-11

**Authors:** Irina Odnoletkova, Dirk Ramaekers, Frank Nobels, Geert Goderis, Bert Aertgeerts, Lieven Annemans

**Affiliations:** 1 Leuven Institute for Healthcare Policy, KU Leuven, Leuven, Belgium; 2 Department of Endocrinology, OLV Hospital Aalst, Aalst, Belgium; 3 Academic Center for General Practice, KU Leuven, Leuven, Belgium; 4 Department of Public Health, Ghent University, Ghent, Belgium; Florida International University Herbert Wertheim College of Medicine, UNITED STATES

## Abstract

**Background:**

People with diabetes have a high risk of developing micro- and macrovascular complications associated with diminished life expectancy and elevated treatment costs. Patient education programs can improve diabetes control in the short term, but their cost-effectiveness is uncertain. Our study aimed to analyze the lifelong cost-effectiveness of a nurse-led telecoaching program compared to usual care in people with type 2 diabetes from the perspective of the Belgian healthcare system.

**Methods:**

The UKPDS Outcomes Model was populated with patient-level data from an 18-month randomized clinical trial in the Belgian primary care sector involving 574 participants; trial data were extrapolated to 40 years; Quality Adjusted Life Years (QALYs), treatment costs and Incremental Cost-Effectiveness Ratio (ICER) were calculated for the entire cohort and the subgroup with poor glycemic control at baseline (“elevated HbA1c subgroup”) and the associated uncertainty was explored.

**Results:**

The cumulative mean QALY (95% CI) gain was 0.21 (0.13; 0.28) overall and 0.56 (0.43; 0.68) in elevated HbA1c subgroup; the respective incremental costs were €1,147 (188; 2,107) and €2,565 (654; 4,474) and the respective ICERs €5,569 (€677; €15,679) and €4,615 (1,207; 9,969) per QALY. In the scenario analysis, repeating the intervention for lifetime had the greatest impact on the cost-effectiveness and resulted in the mean ICERs of €13,034 in the entire cohort and €7,858 in the elevated HbA1c subgroup.

**Conclusion:**

Taking into account reimbursement thresholds applied in West-European countries, nurse-led telecoaching of people with type 2 diabetes may be considered highly cost-effective within the Belgian healthcare system.

**Trial registration:**

NCT01612520

## Introduction

About 387 million people worldwide have diabetes and its prevalence is expected to increase by more than 50% in the coming twenty years [1]. People with diabetes have a high risk of developing cardiovascular disease, kidney failure, neuropathy and retinopathy, that is associated with diminished life expectancy and health-related quality of life and elevated treatment costs [2,3]. About 90% of the diabetes population suffers from type 2 diabetes. Appropriate lifestyle adjustments, medication adherence and regular risk factor control are recommended to achieve sustainable treatment results in type 2 diabetes [3,4]. Patient education aimed at diabetes self-management optimization has been shown to improve diabetes knowledge, self-efficacy and risk factor control [5–7]. However, there is less certainty about its cost-effectiveness [8–10]. The economic evaluations of patient education in type 2 diabetes demonstrated a broad range of results, from cost-saving to not cost-effective at all, while the quality of the studies as well as the underlying clinical evidence varied [11]. Further local context-driven high-quality field research in this area is needed to support policy makers in their choice of appropriate patient support strategies within the budgetary constraints.

The clinical efficacy and cost-effectiveness of diabetes education has not been previously assessed in Belgium. Reimbursed diabetes education was initially introduced in Belgium in 1988 in a hospital ambulatory setting for people with advanced diabetes, i.e. those in need of three or more insulin injections per day. It was extended to primary care in 2009, when “diabetes care trajectories” were launched. Diabetes care trajectories imply that when insulin therapy needs to be initiated, patients are entitled to multidisciplinary care that includes education from a certified diabetes educator and an annual consultation with an endocrinologist, in addition to the regular GP visits. In primary care, diabetes education is mostly delivered in individual face-to-face sessions at the patient’s home and focuses mainly on training in self-administration of insulin and analogues. Most of the non-insulin-dependent patients are currently not offered structured self-management support programs. As individual face-to-face education is costly, alternative delivery modes need to be tested. Telecoaching delivers empowerment and remote support using information and communication technologies. It has the potential to ensure better patient inclusion in diabetes education, while keeping down nurse and patient transport costs.

In 2012, the Independent Health Insurance Fund of Belgium set up a pragmatic randomized clinical trial to investigate the effect of nurse-led telecoaching on diabetes risk factor control among its affiliates with type 2 diabetes. Their claims data were used for the economic evaluation of the intervention. The COACH Program, originally from Australia [12], was tested for the first time in Europe and demonstrated a sustainable improvement in diabetes control. The results of the randomized trial are reported elsewhere [13].

The objective was to analyze the lifelong cost-effectiveness of “The COACH Program”, a nurse-led risk factor target-driven telephone self-management support program compared to usual care, in people with type 2 diabetes in Belgium, from the perspective of the healthcare system.

## Materials and Methods

### Study design

A Markov simulation model with a time horizon of 40 years was populated with patient-level data from a 18-months randomized clinical trial in the Belgian primary care setting involving 574 type 2 diabetes patients [13]. Belgian guidelines for health economic evaluations were followed in methodology and the Consolidated health economic evaluation reporting standards (CHEERS) in reporting [14, 15].

### Intervention

The COACH Program is a risk factor target-driven telephone counselling intervention delivered by diabetes nurse educators, who have followed a one-week training course. It consists of five telephone sessions of 30 minutes on average, spread over 6 months, focused on achieving guideline-recommended diabetes treatment targets through regular control of diabetes risk factors including self-monitoring of blood glucose, appropriate lifestyle adjustments and intensification of medication therapy upon a consultation with the patient’s GP [13].

### Comparator

The comparator was usual care. In Belgium, people with type 2 diabetes are treated by their GPs. When insulin therapy needs to be initiated, the care team is extended by a certified diabetes educator and endocrinologist. Patients with advanced diabetes, in need of three or more insulin injections per day, are usually treated by an endocrinologist-led hospital-based diabetes team.

### Patients

People between the ages of 18 and 75 diagnosed with type 2 diabetes and on diabetes medication therapy were invited into the study by their health insurance fund based on the reimbursement data of glucose-lowering agents in the preceding 12 months. Exclusion criteria comprised patients on corticoid therapy and/or with a debilitating coexisting medical condition such as dialysis, mental illness, or cancer; residence in long-term care facilities; pregnancy; insufficient proficiency in Dutch [13].

### Study horizon

A lifetime prospective modeling was performed with a time horizon of 40 years [16]. In addition, model predictions at time horizons between 1 and 40 years were explored.

### Analytic perspective

The perspective of the Belgian health care system was applied. Direct health care costs to the healthcare system including both the cost for the health insurance and the patient out-of-pocket costs were included. Indirect and/or non-medical costs were not included in the analysis [14].

### Outcome Measures

Incremental cost-effectiveness ratio (ICER), Life Expectancy, Quality Adjusted Life Years (QALYs) and cost of diabetes and its complications. These analyses were performed for the entire cohort as well as for a subgroup of patients with inadequate glycemic control at baseline, i.e. glycated hemoglobin (HbA1c) ≥7%, in line with the clinical trial analysis [13].

### Modeling

The UKPDS Outcome Model was applied for projecting effects observed within the clinical trial over a life-time horizon. It models the occurrence of seven diabetes-related end points: Ischemic Heart Disease (IHD), Myocardial infarction (MI), stroke, heart failure, amputation, End-Stage Renal Disease (ESRD), blindness and death in people with type 2 diabetes to estimate life expectancy, quality-adjusted life expectancy and costs. The model algorithms are based on the observations of the UK Prospective Diabetes Study (UKPDS) participants who were followed up for between six and twenty years [16]. The model uses an integrated system of parametric equations and predicts the annual probability of any of the above end points by using risk factors that include age, sex, ethnicity, duration of diabetes and history of diabetes-related complications, height and weight, smoking status, total cholesterol, HDL cholesterol, systolic blood pressure and HbA1c [17]. The model structure as well as the algorithms for the sequence of events and the parametric equations used within the UKPDS Outcome Model are described in detail elsewhere [16]. The change in the modifiable risk factor values (smoking status, total cholesterol, HDL cholesterol, systolic blood pressure and HbA1c) is modelled based on the observations within the UKPDS study, by predicting the annual point estimates and the associated 95% confidence intervals. The occurrence of events is predicted using Monte Carlo methods [17].

The model has undergone internal and external validation [16]. Developed using data from patients with newly-diagnosed type 2 diabetes, it generated results close to those observed in clinical trials on patients in different stages of type 2 diabetes in a cross-validation exercise [18]. The model is freely available for academic research, as a pre-programmed Excel 2010 file.

### Clinical trial

The randomized clinical trial (RCT) underlying this economic evaluation enrolled 574 Dutch-speaking independently living affiliates of the Flemish Independent Health Insurance Fund “Partena”. Half of them (N = 287) were assigned to the intervention and the other half to the control group. Their median age was 64 years; 62% were men; all were on glucose-lowering medication therapy, of whom 14% on insulin or analogues. The average duration of type 2 diabetes was 7 years; 34% of patients had at least one comorbidity. The mean (SD) baseline HbA1c was 7.0 (1.0) % in all participants and 7.9 (0.9) % in the subgroup with HbA1c≥7% (further “elevated HbA1c subgroup”), Total Cholesterol: 176 (38) mg/dl, Blood Pressure: 133 (17)/75 (10) mmHg, Body Mass Index: 30 (5) kg/m2. All participants have followed a school education, of whom 40% had completed primary, 39% secondary and 21% tertiary education [19]; the majority (64%) were retired [13].

The primary outcome measure was the mean absolute change in HbA1c at 6 months in the entire study group and the elevated HbA1c subgroup at baseline. Secondary outcomes were: change in HbA1c at 18 months; change in total cholesterol, low-density lipoprotein, high-density lipoprotein, triglycerides, blood pressure, body mass index, smoking status, self-perceived health status, at 6 and 18 months’ follow-up.

At 6 months, the mean (95% CI) between-group difference in HbA1c change (%) was -0.2 (-0.3 to -0.1, *P* = .003) overall and -0.4 (-0.7 to -0.2, *P* = .001) in the elevated HbA1c subgroup, in favor of the intervention. Other between-group differences in change were observed at 6 months in BMI (kg/m2): -0.4 (-0.6 to -0.1, *P* = .003) and TC (mg/dl): -6 (-11 to -1, *P* = .012). At 18 months’ follow-up, i.e. 12 months after the completion of the intervention, the improvement in HbA1c was sustained: -0.2 (-0.3 to -0.0, *P* = .046) in the total sample and -0.4 (-0.7 to -0.1, *P* = .023) in the elevated HbA1c subgroup. No other between-group differences were observed at 6 and 18 months’ follow-up.

### Data input

#### Clinical data

The data collected within the clinical trial were incorporated in the model for each patient of both trial arms: age, sex, ethnicity, duration of diabetes, history of diabetes-related complications, height and weight at baseline as well as smoking status, total cholesterol, HDL cholesterol, systolic blood pressure and HbA1c outcomes at all three measurement points (Tables 1 and 2).

**Table 1:** Trial participants’ data incorporated in the UKPDS Model. Baseline characteristics.

Characteristic	Intervention group (n = 287)	Control group (n = 287)
Male, No (%)	173 (60)	180 (63)
Age, years: Median (range)	65.9 (35–75)	63.9 (35–75)
Diagnosis of type 2 diabetes since, No (%)		
≤ 2 years	46 (16)	41 (14)
≥ 10 years	94 (33)	91 (32)
With one or more comorbidities (s), No (%)	92 (32)	103 (36)
Ischemic heart disease	35 (12)	39 (14)
Heart Failure	21 (7)	14 (5)
Myocardial infarction	11 (4)	13 (5)
Stroke	11 (4)	4 (1)

**Table 2:** Trial participants’ data incorporated in the UKPDS Model. Risk factor outcomes at three measurement points.

	Baseline		Year 1		Year 2	
Risk factor, Mean (SD)	Intervention	Control	Intervention	Control	Intervention	Control
HbA1C (%), all	7.0 (1.1)	7.0 (1.0)	6.8 (0.9)	7.0 (1.1)	6.9 (1.0)	7.0 (1.1)
HbA1c (%), subgroup	7.9 (1.0)	7.8 (0.8)	7.4 (0.9)	7.8 (1.1)	7.4 (1.0)	7.7 (1.2)
Weight (kg)	86.1 (16.9)	88.3(16.6)	84.8 (16.4)	87.0(15.9)	85.9 (16.6)	87.3 (15.4)
BMI (kg/m2)*	30.2 (4.9)	30.6 (5.2)	29.6 (4.9)	30.4 (5.1)	29.9 (5.0)	30.4 (5.1)
Total Cholesterol (mg/dl)	173 (37)	178 (39)	165 (36)	176 (39)	162 (34)	170 (49)
HDL-Cholesterol (mg/dl)	52 (16)	51 (14)	53 (15)	53 (16)	52 (15)	52 (15)
Systolic BP (mmHg)	133 (18)	132 (17)	128 (16)	130 (16)	128 (14)	130 (15)
Non-smokers (%)	85.7%	80.7%	87.8%	81.3%	88.6%	84.0%

*Height and weight were required only at baseline and not in the subsequent years.

#### Cost data

The mean annual total healthcare cost in diabetes patients without complications at baseline (95% CI) was €3,921 (3,216; 4,627). It was calculated as a mean of the sum of the healthcare system costs and the legally imposed patient contributions in the subgroup of all trial participants without self-reported comorbidities, in the year prior to the date of the randomization. The claims database of the sickness funds was used as the data source.

The health care costs associated with each fatal or non-fatal diabetes-related complication in the year of the event and in the subsequent years were collected from country-specific published sources [20–27]. All costs were updated to 2013 Euros by using the Belgian health care inflation rates [28] (Table 3).

**Table 3:** Data input in the UKPDS Outcome Model: Treatment costs of diabetes and complications and associated health utilities.

	Fatal (acute)	Non-fatal (at the time of event, acute)	Cost in subsequent years	Utility decrement at diagnosis (event)	Utility decrement in subsequent years
Ischemic heart disease (CHD)	N.A.	€10,976 [20]	€6,044 [21]	-0.09 [30]	-0.046 [33]
Myocardial infarction	€3,829[25;26]	€7,989 [20]	€6,044 [21]	-0.055 [30]	-0.032 [33]
Heart failure	€10,416 [20]	€10,416 [20]	€7,431 [22]	-0.108 [30]	-0.05 [33]
Stroke	€16,658 [22]	€16,658 [22]	€6,030 [21]	-0.164 [30]	-0.061 [33]
Diabetes-related foot amputation	€46,387 [23]	€46,387 [23]	€781 [23]	-0.280 [30]	-0.13 [34]
Diabetes-related blindness	N.A.	€5,382 [24]	5,382 [24]	-0.175 [30]	-0.175 [31]
End stage renal disease	57,078 [25]	57,078 [25]	57,078 [25]	-0.263 [31]	-0.248 [33]

#### Health utilities

The initial utility level, derived from self-reporting of the trial participants using the EQ-5D 3-L questionnaire and calculated as overall sample mean at baseline based on the Flemish utility value system, was 0.785 (0.765; 0.805) [14,29] and did not deviate from the baseline utility level observed within UKPDS. Utility decrements for each of the seven diabetes-related complications at time of event were adopted from the UKPDS Outcome Model and in the subsequent years from other published research that used the same questionnaire, i.e. EQ-5D 3-L and the UK utility value system [30–34] (Table 3).

### Discounting future costs and outcomes

Future costs were discounted at 3.0%, future QALYs gained at 1.5% per annum [14].

### Incremental costs of the intervention

The incremental cost of the intervention was calculated as sum of three components: 1) incremental long-term costs forecasted with the UKPDS Outcome Model, 2) incremental costs in the year of the trial, and 3) costs of the intervention itself.

#### Within-trial incremental costs

At baseline, the annual mean total healthcare cost (CI) was €5,543 (4,410–6,677) in the intervention and €4,101 (3,375–4,827) in the control group and in the year of the trial €5,516 (4,630; 6,402) and €4,757 (3,892–5,622), implying a change of -1% and +16% respectively (Table 4). This inverse trend was closely associated with the change in hospitalization costs (R2 = .930, *P* < .001). As the observed opposite tendency in the change in hospitalization costs (-18.5% in the intervention and +56% in the control group) could have occurred by chance and/ or due to costly hospitalizations not associated with diabetes, the incremental cost calculation was based on the change in the ambulatory costs. Indeed, since the intervention is intended to optimize medical management, it was expected that costs of ambulatory care in the intervention arm would increase during the year of the trial. Such an increase was actually observed and comprised 9% in the intervention group compared to 4% in the control group (Table 3). A more detailed analysis revealed a change in the intervention and control group respectively, in the number of endocrinologist consultations: +35% and -10% (*P* = .023), HbA1c tests: +9% and -12% (*P <* .001), lipid tests: +8% and -16% (*P <* .001) and consumption of lipid modifying agents (measured in number of daily defined doses): +14% and +1% (*P <* .001) [13].

**Table 4:** Ambulatory, hospital and total healthcare costs observed in each trial arm at baseline and in the year of the trial.

Healthcare costs	Intervention group, Mean (CI)			Control group, Mean (CI)		
Year	Baseline	Trial	Change	Baseline	Trial	Change
Ambulatory	3,697(3,106; 4,288)	4,012(3,437; 4,587)	+9.0%	3,148(2,804; 3,492)	3,271(2,909; 3,633)	+4,0%
Hospital	1,846(1,067; 2,626)	1,504(992; 2,016)	-18.5%	953(445–1,461)	1,486(878–2,094)	+56.0%
Total	5,543(4,410; 6,677)	5,516(4,630; 6,402)	-0.5%	4,101(3,375; 4,827)	4,757(3,892; 5,622)	+16.0%

After a regression-based adjustment for the between-group difference in the ambulatory costs at baseline [35], a within-trial ambulatory incremental intervention cost of €270 overall and €179 in the elevated HbA1c subgroup was obtained (Table 5).

**Table 5:** Calculation of the incremental within-trial healthcare costs associated with the intervention, in the entire cohort and the elevated HbA1c subgroup.

Healthcare costs	Total sample		Elevated HbA1c subgroup	
Group	Intervention	Control	Intervention	Control
Ambulatory annual healthcare costs, € (95% CI)				
Trial year, baseline-adjusted*	3,777 (3,499; 4,054)	3,507 (3,304; 3,711)	3,768 (3,410; 4,125)	3,598 (3,310; 3,867)
Incremental intervention costs	270 (395; 343)	N.A.	179 (100; 258)	N.A.

*Regression based adjustment for the between-group difference in baseline costs on the observed data, equation: $HC_{i2}^{Adj}=HC_{i2}-\beta \left(HC_{i1}-\bar{HC_{1}}\right)$, with i = 1,2 being the group indicator and *β* obtained from a regression of *HC*_2_ on *HC*_1_, being the health care cost in the year of the trial and at baseline, respectively) [35]

#### Costs of the intervention

The average operational program cost was €300.3 per patient (Table 6). It consisted of the recruitment costs, fixed costs (software hosting and maintenance) and variable costs (program material: nutrition guide, a tape to measure waist circumference and a set for self-monitoring of blood glucose; patient license fee; actual nurse time spent on coaching and administration; telephone and mailing costs). All costs were registered prospectively during the trial based on the individual time and material registration and the contractual prices. The initial investment costs, such as a 5-day full time nurse training, program translation and technical set-up, are included in the uncertainty analysis and not in the base-case scenario. Allocated to a limited number of patients, the investment costs would skew the per-patient program costs, e.g. divided over 287 trial participants, they amount to €136.85 per patient. However, if 10,000 patients are enrolled, the investment costs per participant decrease to €3.93. Costs imposed by the study that are not part of routine practice, such as protocol-driven nurse assessment visits and laboratory tests, were not included in the analysis.

**Table 6:** Costs of the COACH Program.

Type of costs	Total cost	Costs per patient (N = 287)
Program set-up investment	€39,275.0	€136.85
• Program management training	€14,400.0	
• Program translation	€7,500.0	
• Software configuration	€8,800.0	
• Training of the local coaches	€8,575.0	
Recruitment (mailing to 3115 patients and their GPs)	€3,900.0	€13.6
Fixed costs (software hosting, per year)	€3,790.0	€13.2
Variable costs	€78,494.5	€273.5
• Welcome package		€20.0
• Software license		€50.0
• Nurse time (5.5 hours)		€192.5
• Communication (telephone and mailing)		€11.0
**Total Program costs**	**€86,184.5**	**€300.3**

### Incremental effects of the intervention

Difference in Quality Adjusted Life Expectancy between the intervention and control group with associated 95% confidence intervals was calculated by the UKPDS Outcomes Model and a bootstrap simulation using 999 probability samples (the maximum number of bootstraps programmed within the model).

### Incremental cost-effectiveness ratio

The ICER was calculated as a ratio between the mean incremental costs and the mean incremental QALYs of the intervention group versus controls. The 95% confidence interval of the ICER was calculated by using the upper and lower confidence levels of incremental costs and utilities obtained with the probabilistic sensitivity analysis programmed within the UKPDS Outcomes Model. In addition to the base-case 40 years’ time horizon, ICERs were calculated at 1, 2, 5 years and further at each 5-year interval.

### Handling missing data and analysis of uncertainty

The RCT loss to follow-up at 6 and 18 months was 11% and 16% in the intervention group and 9% and 14% in the control group respectively (13). For the missing clinical data, a single imputation technique was applied using Statistics Analysis System (SAS, version 9.2), i.e. for each of the variables: smoking status, total cholesterol, HDL cholesterol, systolic blood pressure, HbA1c, age and diabetes duration, the mean value was imputed conditional on the other observed values, assuming a multivariate normal distribution. There were no missing claims data.

The parameter—and methodological uncertainty was handled by one-way sensitivity analysis and presented in a Tornado diagram illustrating the impact of different scenarios on the value of ICER. The following scenarios were explored: 1) the program costs varied by 50%; 2) the costs of complications varied by 50%; 3) the upper and lower confidence levels of utility decrements; 4) discount rates for costs and effects set to 0% and to 5%; 5) the effect of the intervention disappearing beyond 18 months, or staying unchanged for lifetime; 6) the intervention repeating bi-annually for 20 years, to sustain the achieved effect.

## Results

**Fig 1:**
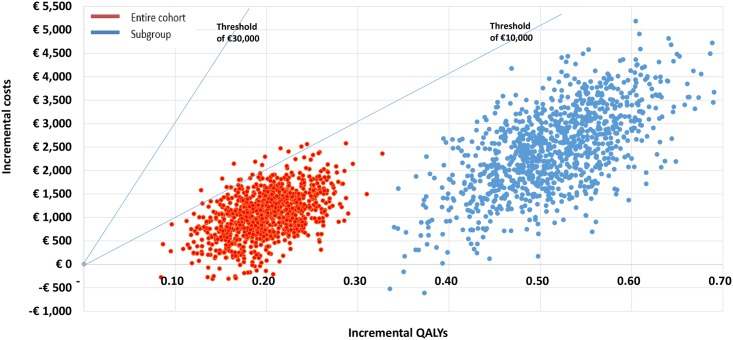
Cost-effectiveness plane based on 999 bootstraps of costs and QALYs. Bootstrapping results of the entire cohort and the elevated HbA1c subgroup, base-case analysis with 40 years’ time horizon.

**Fig 2:**
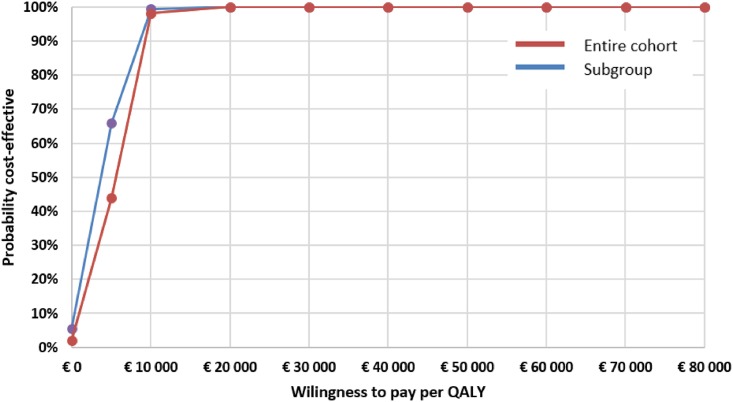
Cost-effectiveness acceptability curves for the COACH Program based on the Monte Carlo simulation of data from the entire cohort and the subgroup with poorly controlled HbA1c at baseline. Baseline analysis with 40 years’ time horizon.

In the basecase scenario analysis of the entire cohort data, the UKPDS Outcomes Model (further “Model”) calculated a mean Life Expectancy (95% CI) of 10.52 (9.61; 11.44) years in the intervention group versus 10.26 (9.36; 11.16) in the control group, corresponding with Quality Adjusted Life Expectancy of 8.04 (7.36; 8.71) versus 7.83 (7.17; 8.49) respectively and implying 0.21 (0.13; 0.28) QALYs gained with the COACH Program. At 40 years’ horizon, the Model forecasted a cumulative decrease in the event rate in the intervention group by 0.2% for IHD, 0.9% for MI, 1.3% for heart failure, 0.8% for stroke and 0.3% for all-cause death (Table 7). The long-term treatment cost of diabetes and complications computed by the Model was respectively €57,226 (50,408; 64,044) and €56,649 (49,939; 63,358). After adding the incremental within-trial costs and the cost of the intervention, the mean total incremental long-term cost in the intervention group was €1,147 (188; 2,107). The mean ICER (95% CI) was €5,569 per QALY (€677; €15,679), with a 2.0% probability that the intervention is cost-saving and a 98.2% probability that the ICER lies below the threshold of €10,000 per QALY (Figs 1 and 2).

**Table 7:** Cumulative event rates from modeling simulation at 5, 10, 20, 30 and 40 years, %.

Complications	Year	Intervention group		Control group		Difference (I-C)	
		Entire cohort	Subgroup	Entire cohort	Subgroup	Entire cohort	Subgroup
Ischemic heart disease	5	1.93	2.13	2.20	2.45	-0.28	-0.32
	10	4.00	4.48	4.51	4.99	-0.51	-0.51
	20	6.57	7.00	6.90	7.30	-0.33	-0.31
	30	7.23	7.62	7.47	7.63	-0.24	-0.01
	40	7.35	7.79	7.52	7.72	-0.17	0.07
Myocardial infarction	5	11.40	13.16	11.42	12.76	-0.02	0.40
	10	19.88	22.07	20.25	21.18	-0.38	0.89
	20	28.20	30.05	29.21	29.55	-1.01	0.51
	30	30.09	32.12	31.08	30.88	-0.99	1.25
	40	30.41	32.46	31.29	30.95	-0.88	1.52
Heart failure	5	3.65	3.98	4.15	4.69	-0.50	-0.71
	10	7.20	7.56	7.99	8.73	-0.79	-1.17
	20	11.39	11.97	12.62	13.17	-1.23	-1.20
	30	12.30	13.03	13.62	13.98	-1.32	-0.95
	40	12.45	13.17	13.73	14.01	-1.28	-0.84
Stroke	5	4.64	5.25	5.06	5.57	-0.42	-0.32
	10	8.63	9.25	9.17	9.99	-0.54	-0.75
	20	12.81	13.38	13.57	14.29	-0.76	-0.90
	30	13.70	14.23	14.53	15.05	-0.83	-0.82
	40	13.79	14.32	14.61	15.08	-0.82	-0.76
Amputation	5	0.32	0.36	0.28	0.44	0.03	-0.08
	10	0.75	1.02	0.70	0.95	0.05	0.07
	20	1.53	1.86	1.52	1.82	0.02	0.04
	30	1.86	2.21	1.78	2.10	0.08	0.10
	40	1.93	2.24	1.84	2.13	0.09	0.11
Blindness	5	2.46	2.88	2.56	2.70	-0.09	0.17
	10	4.51	4.46	4.56	4.67	-0.06	-0.21
	20	6.48	6.15	6.56	6.71	-0.08	-0.57
	30	6.92	6.52	6.98	7.06	-0.06	-0.55
	40	6.95	6.58	7.02	7.12	-0.07	-0.54
Renal Failure	5	0.23	0.28	0.24	0.25	-0.01	0.03
	10	0.62	0.60	0.59	0.63	0.03	-0.03
	20	1.18	1.18	1.15	1.11	0.03	0.08
	30	1.41	1.44	1.40	1.30	0.01	0.14
	40	1.47	1.55	1.45	1.30	0.02	0.25
All cause death	5	27.05	30.10	27.62	31.41	-0.56	-1.32
	10	48.94	52.74	49.43	54.11	-0.50	-1.37
	20	81.60	82.67	82.51	86.73	-0.92	-4.07
	30	95.39	94.90	96.73	98.36	-1.35	-3.46
	40	99.51	99.45	99.81	99.98	-0.30	-0.52

In the elevated HbA1c subgroup, the Model predicted a Life Expectancy of 10.05 (9.15; 10.96) in the intervention and 9.33 (8.47; 10.19) in the control group and the Quality Adjusted Life Expectancy of 7.66 (6.99; 8.33) and 7.10 (6.47; 7.74) respectively, meaning a QALY gain of 0.56 (0.43; 0.68) achieved with The COACH Program. The modeled long-term treatment costs were €55,876 (48,947; 62,805) in the intervention and €53,855 (47,095; 60,614) in the control group, resulting in an incremental total long-term cost of €2,565 (654; 4,474) and an ICER of €4,615 (1,207; 9,969). The probability that the intervention would be cost-saving in people with poorly controlled HbA1c was 0.3%, while the chance that the ICER lies below the threshold of €10,000 per QALY equaled 100% (Figs 1 and 2).

**Fig 3:**
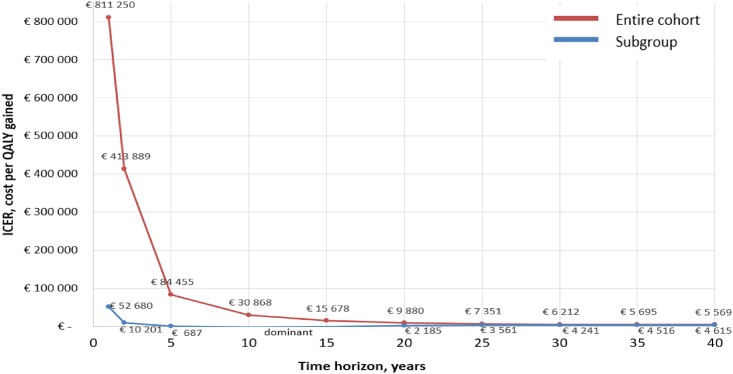
ICER of The COACH Program as a function of the applied time horizon. Results for the entire cohort and the elevated HbA1c subgroup.

An inverse relationship was observed between the ICER values and the applied time horizon in the entire cohort, with €811,250 per QALY in the first year after the program delivery and a steep fall to €84,455 in year five; €30,868 in year ten; €9,880 and €6,212 in year twenty and thirty respectively. In the elevated HbA1c subgroup, the ICER was €52,680 per QALY in the first year after the trial, €10,201 in the second year, and did not exceed the value of the 40-time horizon, at any of the other simulated years (Fig 3).

**Fig 4:**
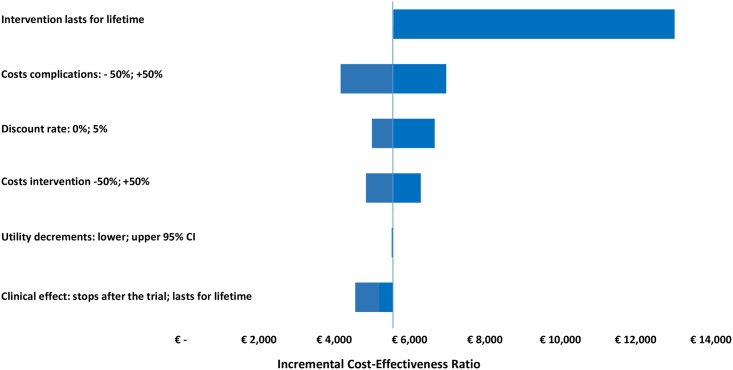
One-Way Sensitivity Analysis showing the influence of changing different parameters on the long-term cost-effectiveness in the entire cohort.

**Fig 5:**
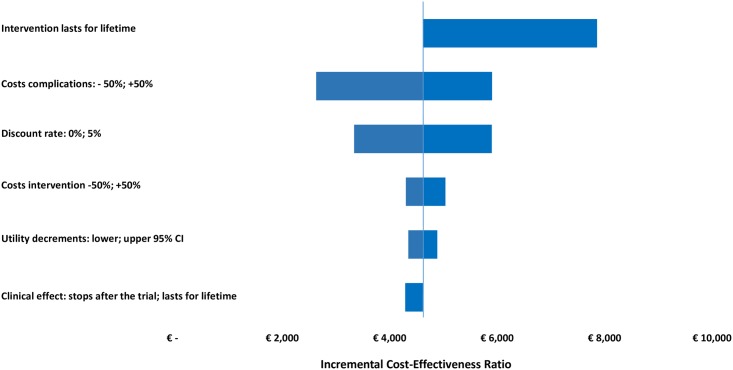
One-Way Sensitivity Analysis showing the influence of changing different parameters on the long-term cost-effectiveness in the subgroup with poorly controlled HbA1c at baseline.

The one-way sensitivity analysis demonstrated a lifetime QALY gain in the intervention group in all applied scenarios, with a variability of the ICER between €4,168 and €13,034 per QALY in the entire cohort and between €2,629 and €7,858 per QALY in the elevated HbA1c subgroup. Assuming that the effect of the intervention disappeared after 18 months, the calculations showed a mean incremental QALY of 0.14 in the entire cohort and 0.53 in the elevated HbA1c subgroup, with the respective ICERs of €4,556 and €3,336 per QALY. Assuming that the effect stayed unchanged for life, the QALY gained with telecoaching were 0.32 and 0.67, and the ICERs €5,198 and €5,586 per QALY, respectively. The hypothesis that the intervention needs to be repeated bi-annually for life, to sustain the achieved effect, had the greatest impact on the cost-effectiveness, followed by varying the cost of diabetes complications (Figs 4 and 5).

## Discussion

The cost-effectiveness analysis of The COACH Program adapted to the Belgian primary care setting and performed by populating the UKPDS Outcomes Model with the data of the randomized clinical trial participants, showed a mean QALY gain of 0.21 in the entire cohort and 0.56 in the subgroup with poorly controlled HbA1c at baseline. The mean ICER in the respective study cohorts was €5,569 and €4,615 per QALY, with 2.0% and 0.3% respective probabilities for telecoaching to be cost-saving and 98.2% and 100% probabilities that the value of ICER lies below the threshold of €10,000 per QALY. In the intervention group, a gain in QALYs was demonstrated in all scenarios and was associated with a greater/ longer healthcare utilization. The assumption that the telecoaching program needs to be repeated bi-annually for life, to sustain the achieved effect, had the greatest impact on the ICER: €13,034 and €7,858 in the entire cohort and the elevated HbA1c subgroup respectively.

Though the application of a single ICER threshold in the national reimbursement decisions is not common [36], the World Health Organization recommends considering health technologies with the ICER below the value of the gross domestic product (GDP) per capita as very cost-effective [37]. With the GDP above €43,000 [38], the target-driven nurse-led telecoaching of people with type 2 diabetes has great potential to be considered cost-effective within the Belgian healthcare system.

The results are comparable with those obtained in the cost-effectiveness study of diabetes group education in the UK, where a lifetime prediction with the Sheffield diabetes model was applied and the ICER of £5,387 (€6,700) per QALY was reported [39]. No other similar European studies were identified. Several published economic evaluations applied a short-term analytic horizon, which complicated interpretation of the results [40–42]. The meaningfulness of short-term cost-effectiveness analysis of diabetes self-management programs is questionable, as it has been shown that the greatest costs occur in the year of the delivery and decrease in subsequent years, while most of the benefits occur after several years of follow-up [43]. The analysis of ICER in the entire cohort at different time points confirmed this observation, showing a consistent improvement in the cost-effectiveness over time. A key question for policy makers therefore is whether they are prepared to consider a longer term horizon in their decision-making [44].

While the study aimed to assess the long-term cost-effectiveness, the results of the within- trial analysis deserve attention. Though participants of the telecoaching program demonstrated an increase in utilization of the guideline-recommended ambulatory diabetes care, the overall healthcare costs in the year of the trial decreased by 1% in the intervention group. The control group showed the opposite trend: a decrease in the guideline-recommended care consumption and an increase in overall healthcare costs by 16%. Change in total costs in both groups was strongly associated with hospitalization costs; however, it was difficult to attribute these changes to the intervention due to a lack of insight into the admission causes. Diabetes education has previously been shown to have a positive impact on the number of hospital admissions in the short to medium term, based on retrospective studies [45,46],—a hypothesis that requires further testing in a randomized setting.

Every health economic model has its limitations. Differences between patient baseline characteristics and the clinical setting underlying the model, and those used to populate the model, may result in varying long-term disease progression patterns [47]. However, given the scarcity of appropriate data and resources, it is not feasible to develop new models specific to each setting [48]. From at least thirteen available predictive diabetes models, the UKPDS Outcomes Model has undergone the most extensive external validation of its ability to predict the incidence of cardiovascular disease through a comparison with the results of large cohort studies in people with type 2 diabetes. It has been shown to consequently overestimate the risk of coronary heart disease and stroke [48–52]. The Model should therefore be used with caution for the prediction of the absolute risk of diabetes complications, but is believed to provide a reasonable prediction of the incremental event rate and be a suitable method for resource prioritization [50–52]. The Model performance in estimating the risk of microvascular complications should be further investigated. Introduction of electronic patient records at the national level and their structural use in epidemiological research should be of great importance for the development of well-performing models in different patient subgroups. Recently, the second version of the UKPDS Outcomes Model was released; however, the validation of its equations is still ongoing [53].

Until now, most nurse-led telecoaching programs have been unsuccessful in improving glycemic control, even though the recruited patients had an elevated HbA1c at baseline [54–56]. The COACH Program implemented in the Belgian primary care setting resulted in a clinically modest HbA1c reduction by 0.2% in the total sample and a clinically significant reduction by 0.4% in the subgroup of patients with HbA1c≥7% at baseline, sustainably lowering the mean HbA1c in the intervention group to the recommended target below 7%. In addition, clinically modest improvements in BMI and Total Cholesterol were observed at 6 months’ follow-up. A comparable effect on the glycemic control was achieved with face-to-face and group education programs; however, it was limited to the subgroups with HbA1c ≥ 8% at baseline [5], and tended to disappear within one to three months after the completion of the intervention [6]. The concept of the COACH Program therefore merits special attention. In the past 15 years the COACH Program has proved effective in different conditions and settings [12,13,57]. The critical success factors of the program, and tailored session content focused on patient empowerment; goal-setting and negotiation of an action plan that includes appropriate medical visits, lifestyle adjustments and medication adherence, quality assurance measures and a constructive interaction with the involved physicians, have to be considered in the implementation phase [13].

Limitations to the overall generalizability of the study results include possible positive self-selection of patients recruited into the study, exclusion of people with debilitating medical conditions and the Belgian-specific cultural, organizational and economic context. However, considering a fair heterogeneity of the study population and the pragmatic nature of the clinical trial, the results of the study are potentially transferrable to primary care settings in Western countries. The baseline characteristics of the trial participants were comparable to those of the general Belgian population with type 2 diabetes in terms of clinical, biomedical and demographic data [58,59]. Moreover, a similar clinical effect was achieved with The COACH Program in patients with type 2 diabetes in different cultural contexts [13,57]. Further research should identify those groups of patients who might benefit from diabetes education through alternative delivery modes. Socially disadvantaged people and those with limited language skills may present particular challenges to goal-based care and require more intensive modes of support [13].

More economic evaluations of healthcare programs are needed to support the policy makers in their decisions on budget allocation. Currently, patient self-management support programs are structurally underfinanced, while relevant health economic research is strongly underrepresented compared to evaluations of medicines. However, the results of the cost-effectiveness analyses are not the only criterion worth considering when making reimbursement decisions [60]. Severity of disease, size of the target population, budget impact, and availability of treatment alternatives may also play a role alongside legal, ethical and organizational issues [61]. At present, reimbursement decisions in healthcare frequently lack a systematic approach [62]. Policy tools such as priority setting and multi-criteria decision-making are being explored and have the potential to increase the transparency of reimbursement decisions [60, 63–65].

## Abbreviations

BMI: Body Mass Index
BP: Blood Pressure
CHEERS: Consolidated health economic evaluation reporting standards
ESRD: End-Stage Renal Disease
HbA1c: Glycated hemoglobin
HDL: High-density lipoprotein
ICER: Incremental Cost-Effectiveness Ratio
IHD: Ischemic Heart Disease
MI: Myocardial Infarction
QALY: Quality Adjusted Life Years
RCT: Randomized Controlled Trial
SAS: Statistics Analysis System
UKPDS: UK Prospective Diabetes Study
